# Improving methane production in cow dung and corn straw co-fermentation systems via enhanced degradation of cellulose by cabbage addition

**DOI:** 10.1038/srep33628

**Published:** 2016-09-19

**Authors:** Wenyang Wu, Yong Chen, Shah Faisal, Aman Khan, Zhengjun Chen, Zhenmin Ling, Pu Liu, Xiangkai Li

**Affiliations:** 1 MOE Key Laboratory of Cell Activities and Stress Adaptations, School of Life Sciences, Lanzhou University, Lanzhou, Gansu, P.R. China

## Abstract

The effects of cabbage waste (CW) addition on methane production in cow dung and corn straw co-fermentation systems were investigated. Four experimental groups, each containing 55 g of substrate, were set up as follows: 100% cow dung (C); 36% cabbage and 64% cow dung (CC); 36% straw and 64% cow dung (SC); and 18% cabbage, 18% straw, and 64% cow dung (CSC). After seven days of fermentation, the maximum methane yield was 134 mL in the CSC group, which was 2.81-fold, 1.78-fold, and 1340-fold higher than that obtained in the CC, SC, and C groups, respectively. CW treatment of the CSC group enhanced cellulase activity and enriched culturable cellulose-degrading bacterial strains. Miseq sequencing data revealed that the predominant phylum in the CSC group was *Bacteroidetes*, which contains most of the cellulose-degrading bacteria. Our results suggested that CW treatment elevated cellulose degradation and promoted methane production.

Cabbage is the most important and popular vegetable in China. The production of cabbage is three million tons per year. Surprisingly, it is reported that 30% of the total production is discarded as waste^1^. Currently, landfills are the most common way for cabbage waste (CW) treatment^2^. CW is easily degraded, leading to the generation of acetic acid by microorganisms. This process decreases the pH of soils, making them incompatible for the growth of vegetation^3^. Previous studies have also reported that combustion is a conventional approach for CW disposal^4^; however, most CW has a water content of nearly 90%. Incomplete combustion generates toxic gases such as sulfur dioxide (SO_2_) and sulfur monoxide (SO), which are harmful to the environment^5^. An eco-friendly and cost-efficient mechanism of CW disposal is therefore needed.

Previous studies reported that anaerobic digestion (AD) is a promising disposal approach, as CW can provide sufficient cellulose and water for digestion systems^6^. In light of the conventional energy crisis, AD not only remediates cabbage waste but also provides renewable energy^7^. It has been reported that the optimum cellulose content for AD is 30%^8^, but CW consists of nearly 90% water and only 9% cellulose. Such low cellulose content limits hydrolysis by AD. The rate of hydrolysis is one of the key factors affecting fermentation efficiency^8^,^9^. Previous studies have shown that corn straw, which contains high quantities of cellulose (43.5%, dry basis), is a good provider of cellulose for AD^10^.

Hence, we propose that CW treatment will improve methane production in corn straw and cow dung co-AD systems. In this study, CW was added to fermentation systems, and the methane yield was 134 mL after 7 days fermentation, which represents the best methane production efficiency compared with systems using other substrates.

## Results and Discussion

### Biogas volume and methane content

**Figure 1:**
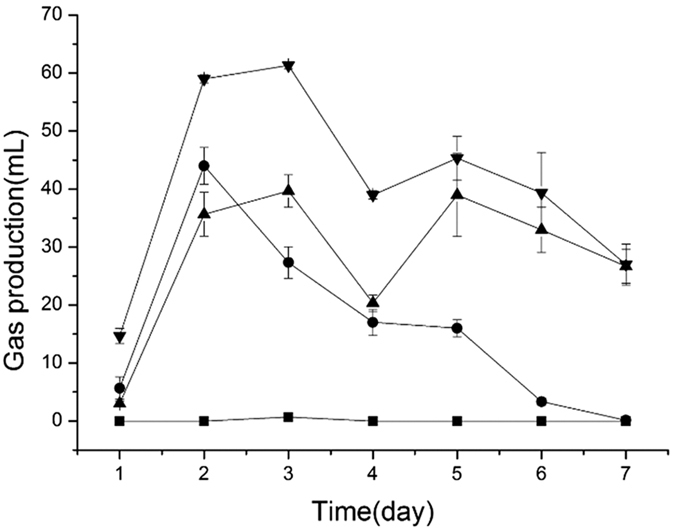
Daily biogas production for different groups. (▴) SC, 64% cow dung and 36% straw after 7 days fermentation; (⦁) CC, 64% cow dung and 36% cabbage after 7 days fermentation; (▾) CSC, 64% cow dung, 18% cabbage, and 18% straw after 7 days fermentation; (◾) C, 100% cow dung after 7 days fermentation.

**Figure 2:**
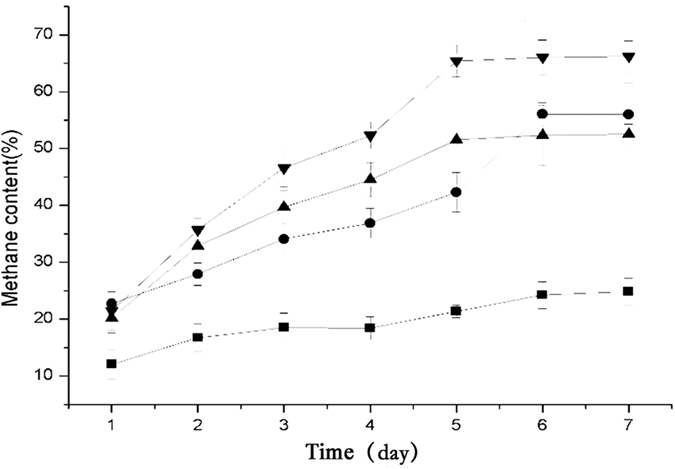
Methane content for different groups. (▴) SC, 64% cow dung and 36% straw after 7 days fermentation; (⦁) CC, 64% cow dung and 36% cabbage after 7 days fermentation; (▾) CSC, 64% cow dung, 18% cabbage, and 18% straw after 7 days fermentation; (◾) C, 100% cow dung after 7 days fermentation.

After 7 days fermentation, the final pH of all groups ranged between 5.8 and 6.3. Total solids (TS) and volatile solids (VS) of each groups were shown (Table 1). The total biogas volumes of groups C (100% cow dung), SC (36% straw and 64% cow dung), CC (36% cabbage and 64% cow dung) and CSC (18% cabbage, 18% straw, and 64% cow dung), under 30 °C, were 0.6 mL, 197 mL, 113 mL and 266 mL, respectively, while the average methane proportions of biogas were 17%, 39.4%, 42.1% and 50.4%, respectively (Figs 1 and 2; Table S1). Methane generation in the C, SC, CC, and CSC groups was 0.053 mL/g-VS, 3.704 mL/g-VS, 9.207 mL/g-VS, and 14.094 mL/g-VS, respectively (Table 1). These results suggested that CW treatment significantly promoted methane production. It is reported that using food wastes and straw as co-substrates increases methane production by 149.7% compared to individual straw AD with 612 g substrates^11^. The composition of CW is similar to that of food wastes, particularly in terms of C/N ratio and water content. Our data were in agreement with a previous study showing that CW addition elevated methane production^11^. The control C group only produced 0.053 mL/g-VS methane because of a lack of cellulose, which was in agreement with another previous study^12^.

The C/N ratios of the C, SC, CC, and CSC groups were 8.4, 56.4, 18.9, and 30.2, respectively (Table 2). A previous study reported that the optimum C/N ratio in fermentation systems is between 20 and 35^13^. The C/N ratio in the CSC group, which had the best methane production efficiency among all groups, was 30.2. Thus, this result was consistent with the previous study^13^. In addition to the C/N ratio, water content is also an important parameter. C, CC, and CSC groups had 95%, 88%, and 75% water content, respectively (Table 2). A previous study showed that the water content of substrates should be kept at approximately 90% of the total content. Low water content will lead to acetic acid accumulation, which inhibits the fermentation process, and high water content reduces methane production efficiency^14^. Although the substrates of the C, CC, and CSC groups had optimum water content, only the CSC group had high methane production efficiency. Therefore, the CSC group had sufficient cellulose for AD, and CW treatment might have increased cellulase activity.

Straw is characterized by a high percentage of cellulose^15^. However, straw as a single substrate in the SC group showed a methane yield of only 77.6 mL (Table S1). A previous study showed a methane yield of 120 mL using straw as substrate after water addition, which was higher than in the SC group, but lower than the 134.1 mL yield of the CSC group^16^ (Table S1). These results suggest that both, the cellulose and water content from CW, were important for fermentation. The cellulose decomposition rate may be increased after CW addition.

Previous studies reported that pre-treatment of corn straw enhances the hydrolysis step as well as improves the rate of methane production during fermentation^17^. Some biological, chemical, and physical methods have been applied to improve fermentation yields^18^,^19^. In this study, CW addition promotes methane production in cow dung and corn straw co-fermentation systems, which is more convenient than using pre-treatment methods in industrial applications.

### Scanning electron microscopy showed that CW addition increased cellulose decomposition rate

**Figure 3:**
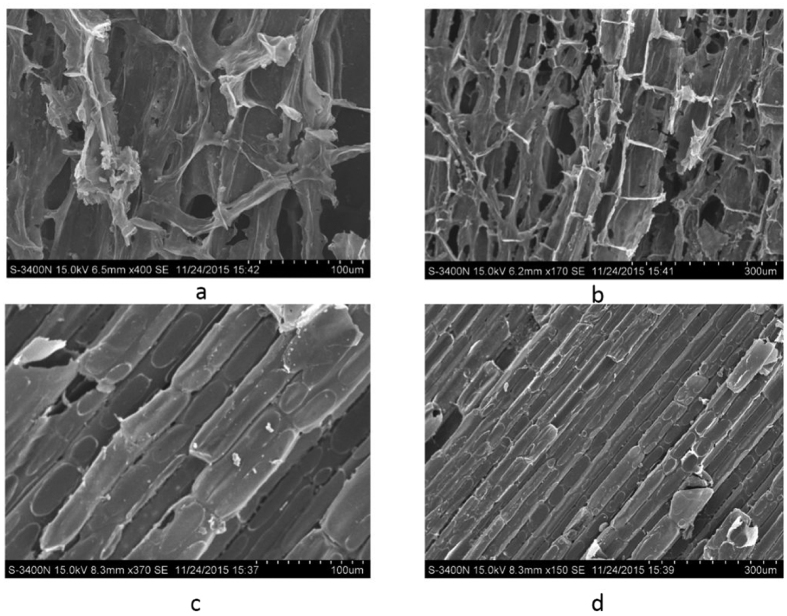
Structure of straw after 7 days fermentation. (**a**) The straw from CSC at a magnification of 400×; (**b**) the straw from CSC at a magnification of 150×; (**c**) the straw from SC at a magnification of 400×; (**d**) the straw from SC at a magnification of 150×.

Scanning electron microscopy (SEM) was used to analyze straw fiber from the SC and CSC groups at a magnification of 400× and 150×, respectively, after 7 days of fermentation. The fiber and texture of straws from the CSC group was completely disrupted. Compared with the CSC group, the structure of straws from the SC group was relatively intact (Fig. 3). These results suggested that the degree of corn straw degradation in the CSC group was higher than that of the SC group.

A previous study reported that the degradation rate of cellulose is the key factor in AD^20^. However, it is difficult to directly convert cellulose to methane without high bacterial cellulase activity^21^. Thus, SEM results suggested that CW addition increased the cellulose decomposition rate and that cellulase activity was enhanced by CW treatment.

### Cellulase specific activity was increased by CW addition

**Figure 4:**
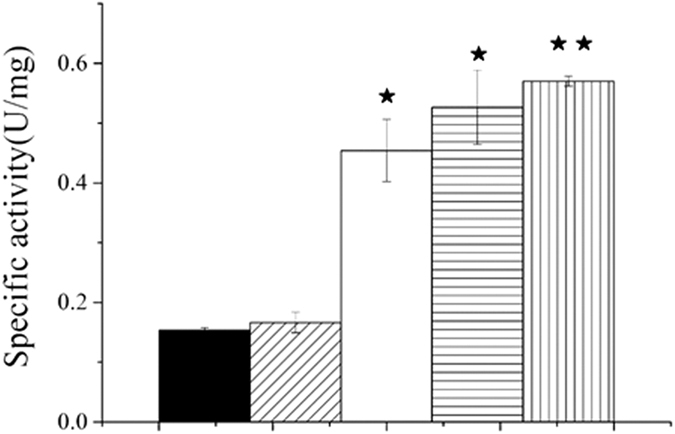
Cellulase specific activity in different groups. (◾) UC, unfermented cow dung; (▤) SC, 64% cow dung and 36% straw after 7 days fermentation; (◽) CC, 64% cow dung and 36% cabbage after 7 days fermentation; (▥) CSC, 64% cow dung, 18% cabbage, and 18% straw after 7 days fermentation; (▨) C, 100% cow dung after 7 days fermentation. Values for the CC, SC and CSC groups that are significantly different from the UC values are indicated by ^★^*P* < 0.05 and ^★★^*P* < 0.01 (n = 3).

To determine the influence of CW addition on cellulase activity, the cellulase enzymatic activity was measured in the different groups. Standard curves of glucose levels and BSA concentrations were created using the DNS method and a TaKaRa Bradford protein assay kit, respectively (Figure S1). As shown in Fig. 4 and Table S2, the cellulase specific activity of the CSC group was 0.57 U/mg (*P*  < 0.01), higher than in the CC and SC groups, which were 0.43 U/mg (*P * < 0.05) and 0.42 U/mg (*P *< 0.05), respectively.

It has been suggested that the surface of straw is the most vital factor in AD, because of its influence on the cellulose hydrolysis rate^22^,^23^. The fact that the highest cellulase specific activity was observed in the CSC group indicates that CW has a relatively susceptible surface. However, CW as the sole substrate yielded a low fermentation efficiency and only 0.43 U/mg cellulase activity, because the acidification of CW after fermentation led to low pH that negatively affected enzyme activity^24^. In the CSC group, CW was shown to decrease the rate of acidification, leading to the maximization of productivity^25^. The cellulase specific activity of the SC group was lower than that of the CSC group. We concluded that this was because of water content insufficiency, hindering the enzyme activity, and that CW can provide sufficient cellulose to co-digestion systems.

### Isolation and identification of cellulose-utilizing bacteria

To determine whether culturable cellulose-utilizing bacteria were enriched by CW treatment, we isolated them from the system. Two cellulose-utilizing strains from unfermented cow dung were isolated. Both isolates can grow using filter paper as the sole carbon source. Based on 16S rRNA sequence similarities, both strains were related to *Bacillus* (Figure S2).

**Figure 5:**
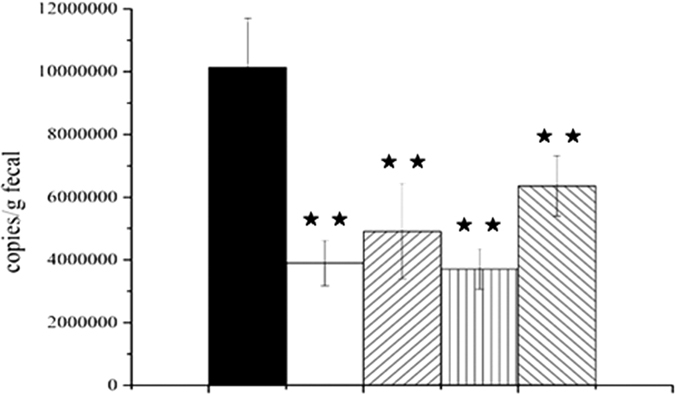
Quantification of cellulose-utilizing bacteria in the fecal microbiota (copies/g of fecal). (◾) UC, unfermented cow dung; (◽) SC, 64% cow dung and 36% straw after 7 days fermentation; (▨) CC, 64% cow dung and 36% cabbage after 7 days fermentation; (▧) CSC, 64% cow dung, 18% cabbage, and 18% straw after 7 days fermentation; (▥) C, 100% cow dung after 7 days fermentation. Values for the C, CC, SC and CSC groups that are significantly different from the UC values are indicated by ^★^*P* < 0.05 and ^★^*P* < 0.01 (n = 3).

The richness of these strains in the different groups was investigated by qRT-PCR method^26^. Standard curves of the optimized assays were created using SYBR Green II (Figure S3). No primer dimers with lower Tm values were observed. The value obtained for strains from the C group was only 3,767,574 copies/g. Number of copies of strains in the SC and CC groups were 3,694,356 (*P* < 0.01) and 4,754,758 (*P* < 0.01) copies/g, respectively. A statistically significant increase was detected in the CSC group compared with the others groups, with 6,380,984 copies/g (*P* < 0.01) (Table S2). These results indicated that CW promoted the growth of cellulose-utilizing strains from cow dung in cow dung and corn straw co-fermentation systems (Fig. 5).

Previous studies have shown that the utilization of cellulose was a key process in methane fermentation and that some *Bacillus* spp. have strong ability to degrade cellulose^27^. In the present study, cellulose-utilizing strains quickly adapted to the new environment following the addition of CW and straw to the fermentation systems, utilizing cellulose as a carbon source. We propose that the composition of the CW and straw mixture, i.e., cellulose and water contents, have contributed to the growth of cellulose-utilizing strains.

### CW addition increased the diversity of *Bacteroidetes* and *Firmicutes*

Previous experiments showed that CW treatment increased culturable cellulose-degrading bacterial strains. Therefore, we used meta 16S sequencing to study the microbial community’s structure after CW treatment. The pyrosequencing reads were clustered and assigned to respective taxonomic branches. 1525 OTUs were obtained from fifteen samples with an average of 1525 ± 87 per sample. Shannon and rarefaction diversity curves revealed that most of the diversity was captured (Figure S4). The richness and α-diversity of the fifteen samples were calculated using observed OTUs (Table S3). UniFrac-PCoA and heatmap results showed that the microbial structures of all groups were different from unfermented cow dung (UC) after 7 days of fermentation (Figures S5 and S6).

**Figure 6:**
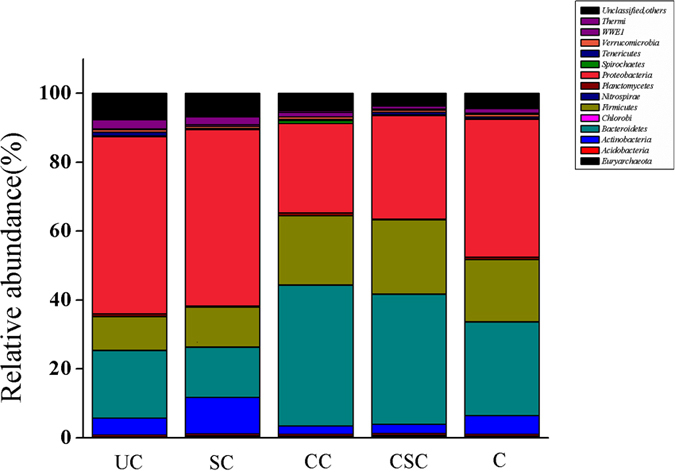
Relative abundance (% of total reads) of bacterial 16S rRNA gene at phylum level.

The bacterial community structure of all groups was composed of 15 phyla (>0.5% level of taxa identified) (Table S4). A major proportion of them were *Proteobacteria*, *Bacteroidetes*, and *Firmicutes* (Fig. 6), which were also reported by^28^,^29^. In our study, *Bacteroidetes* and *Firmicutes* were enriched after CW addition, whereas the richness of *Proteobacteria* decreased. *Bacteroidetes*, which contains most of the cellulose degradation bacteria^30^, was the most significant phylum in the CSC group. In this study, the richness of *Bacteroidetes* increased in the CSC group compared with the UC group. A previous study reported that microorganisms from the *Firmicutes* phylum have the ability to metabolize a variety of substrates including sugars and lignin in acidogenic reactors, which are the main components of CW^31^. Our results were in agreement with this observation as the richness of *Firmicutes* in the CSC group increased by nearly 60% after CW addition, compared with the SC group. These results suggested that the CW treatment have changed the microbial community structure in the fermentation systems.

## Conclusions

CW increases the rate of cellulose degradation and methane production efficiency, yielding 0.57 U/mg cellulase specific activities and generating 134 mL methane over 7 days of fermentation, using 55 g of substrate. *Bacteroidetes* and *Firmicutes* are two of the main phyla involved in anaerobic digestion; their richness increase after CW addition. Based on the above results, utilization of CW in cow dung and corn straw co-fermentation systems is an ideal solution for both, managing CW pollution and partly solving the energy crisis.

## Methods

### Anaerobic co-digestion process

Cow dung was collected from Zhuangyuan Dairy Factory (Lanzhou, China) in May 2014. Straw and cabbage waste (CW) were collected from a farmyard in June 2014. The coordinates of the collection site are 104.09°N, 35.87°E.

Anaerobic digestion reactors (500 mL) were loaded proportionally with mixtures of cow dung, straw and CW. All treatments were conducted in triplicates, and the total mass of cow dung in every treatment was 35 g. Increasing proportions of CW were added, with 0%, 36.4% and 18.2% of total substrate mass in the SC, CC, and CSC groups, respectively. The composition of the UC, C, SC, CC, and CSC groups are shown in Table 2. The initial pH of all groups was 7.2. All reactors were sealed with a rubber stopper that had an outlet to collect biogas and were incubated at 30 °C for seven days. Biogas was collected in a collection bag, and daily methane production volumes were analyzed by gas chromatography (GC) (Agilent Technologies, 7890A, Wilmington, DE, USA)^32^.

Total solids (TS) and volatile solids (VS) were analyzed by APHA standard methods according to a previous study^33^.

### Scanning electron microscopy

The straw structures from SC and CSC after seven days of fermentation were assessed by scanning electron microscopy (SEM). All straw samples (5 g) were first freeze-dried for 16 h and then observed on a Model S-3400N scanning electron microscope (Hitachi, Japan) following metal spraying and fixation of samples on a thin gold layer^34^.

### Determination of cellulase specific activities

Cellulase activity was measured using the DNS (3,5-dinitrosalicylic acid) method^35^. Briefly, 10 g of cow dung was incubated in 50 mM Tris-HCl (pH 8.0) for 15 min at 50 °C. The reaction was then stopped by addition of DNS solution. The treated samples were boiled for 10 min and cooled in icy water to stop the color development, and the optical density was measured at 540 nm using a spectrophotometer. Glucose was used to generate a standard curve using the above method, and cellulase activity was calculated based on that calibration curve. One unit of enzyme activity corresponded to the amount generating the release of 1 μM of glucose per minute.

Cow dung (10 g) was centrifuged at 4000 *g* for 8 min, and the precipitates were washed three times in 0.01 M phosphate buffer (pH 7.0). The precipitates were re-suspended in the same buffer with ultrasonic shaking for 5 min at 0 °C and centrifuged at 20 000 *g* for 25 min at 4 °C. Supernatant protein concentrations were estimated using the Lowry method^36^. The specific activity of cellulase was determined by calculating the ratio of cellulase activity to protein concentration.

### Isolation of cellulase-producing microorganisms

To isolate cellulose-utilizing strains, modified RGCA medium was prepared as follows (per liter): 3 g Na_2_HPO_4_, 3 g NaH_2_PO_4_, 6 g NaCl, 6 g (NH_4_)_2_SO_4_, 0.6 g MgSO_4_, 0.8 g CaCl_2_, 20 mL mineral solution, and 20 mL vitamin solution. Cow dung (1 g) was added to 8 mL 0.85 mmol/L NaCl and incubated at 25 °C for 4 h. This suspension (100 μL) was added to 5 mL modified RGCA medium using filter paper as the only carbon source, and incubated with 180 rpm agitation at 30 °C, for 2 weeks. The strains were incubated on a solid modified RGCA medium containing sodium carboxymethylcellulose at 30 °C for 24 h. Single colonies were collected and re-selected using the above methods.

### Identification of cellulose-utilizing strains

Isolated strains were grown in 10 mL Luria-Bertani liquid medium with 180 rpm agitation at 30 °C, for 24 h. The bacterial DNA was extracted using the MiniBEST Bacterial Genomic DNA Extraction Kit (TaKaRa, Japan). We used the universal primers of the 16S rRNA gene to amplify the 16S rRNA gene fragments. The resulting fragments were sequenced by Shanghai Majorbio Bio-pharm Technology Co. Ltd (China), and compared in the NCBI (http://www.ncbi.nlm.nih.gov/) and EzTaxon (http://www.ezbiocloud.net/) databases.

### PCR amplification assays, purification and cloning of PCR products

The primer to the 16S rRNA gene of the cellulose-utilizing species was designed using PRIMER 5. The sense primer was 5′-TGCCTGTAAGACTGGGATAACT-3′, and the antisense primer was 5′-GTTTACGGCGTGGACTACC-3′. The annealing temperatures and procedures followed for the PCR amplifications were determined in a previous study^37^. These conditions were used to quantify the copies of cellulose-utilizing bacteria from the cow dung samples. The purified products of 16S rRNA were used for cloning and then subsequently ligated into the pGEM-T Easy Vector (Promega Corporation, Madison, WI, USA) according to manufacturer’s specifications. The pGEM-T Easy Vector with the gene products was used to transform *Escherichia coli* DH5α cells using the methods described in a previous study^38^.

### Calculation of 16S rRNA gene copy numbers and standard curves

Plasmid DNA was extracted using Plasmid Mini Kit I (OMEGA). 16S rRNA gene copy numbers in the plasmid DNA were determined following the procedure developed in a previously study^39^. The standard curve was established by quantitative real-time PCR, and generated from a 10-fold dilution series into ddH_2_O. The standard curve of Ct values from DNA samples with known number of copies was used for the quantification process^38^.

### Quantitative real-time RT-PCR

Quantitative real-time PCR was used to determine copy levels of the cellulose-utilizing strains. The 10 μL reaction mixture was composed of 1 μL DNA, 1 μL DNA polymerase, 2.8 μL distilled water, 5 μL SYBR green PCR Master Mix, and 0.2 μL of each primer (20 mmol/L). The cycle had been determined in a previous study^40^. 16S rRNA gene copies per g of wet weight of cow dung were used to express the results.

### Total DNA extraction

The cow dung from the different groups (0.2~0.5 g weight) was removed by centrifuging at 12,000 *g* for 20 min at 4 °C. The samples were washed twice using 1.5 mL of TENP buffer. After vortexing for 10 min, the cow dung was collected by centrifugation (12,000 *g* for 8 min). The E.Z.N.A.TM Soil DNA Kit (Omega Bio-Tech, Inc., USA) was used according to the manufacturer’s instructions. DNA was dissolved in 40 μL ddH_2_O at the final step. Finally, total DNA yield and quality were determined by NanoDrop 3300 (Thermo Fisher Scientific Inc. USA). The extracted DNA was stored at −40 °C for further use.

### Illumina-MiSeq sequencing

The microbial community structure of the different groups and unfermented cow dung (UC) was determined by pyrosequencing^41^. The primers 515F (5′-GTGCCAGCMGCCGCGGTAA-3′) and 806R (5′-GGACTACHVGGGTWTCTAAT-3′) with 10 nt barcodes were used to amplify the V4 hypervariable regions of the 16S rRNA genes. The PCR mixture (25 μL) contained 1.5 mM MgCl_2_, 1 μL buffer, each deoxynucleoside triphosphate at 0.4 μM, each primer at 1.0 μM, 0.5 U of TransStart Fast Pfu DNA Polymerase (TransGen, Biotech, China), and 10 ng of soil genomic DNA. The PCR amplification program included an initial denaturation for 5 min at 94 °C, followed by 38 cycles of 50 s at 94 °C, 70 s at 58 °C, and 60 s at 70 °C, and a final extension step lasting 15 min at 70 °C. Two PCRs were carried out for each sample and combined together following the amplification. The quality of the PCR products was assessed by electrophoresis using 1.0% agarose gel. The correct bands were excised and purified using a Gel Extraction Kit (Omega Bio-tech, USA) and quantified with a Nanodrop. All samples were linked up with equal molar amounts from each sample, and prepared using a TruSeq DNA kit, according to the manufacturer’s instruction, before being sequenced by the Illumina Miseq system using the Reagent Kit v2 2 × 250 bp. The data were analyzed by QIIME Pipeline-Version 1.7.0 (http://qiime.org/tutorials/tutorial.html). Shannon’s diversity index, chao 1 of richness, and UniFrac metrics were calculated.

### Statistical analysis

All the data were subjected to ANOVA tests to determine whether the observed differences between the experimental groups and the control group were significant^42^.

## Additional Information

**How to cite this article**: Wu, W. *et al*. Improving methane production in cow dung and corn straw co-fermentation systems via enhanced degradation of cellulose by cabbage addition. *Sci. Rep.* **6**, 33628; doi: 10.1038/srep33628 (2016).

## Supplementary Material

Supplementary Information

## Figures and Tables

**Table 1:** Summary of performance parameters in the different groups.

Sample ID	Total solids (TS) (%)	Volatile solids (VS) (%)	VS/TS (%)	MPY^a^(ml)	MPY (ml CH_4_/g-VS)
UC	5 ± 0.3	3.4 ± 0.6	67.9 ± 1.4	—	—
SC	54 ± 1.6	45.9 ± 1.5	85 ± 2.5	77.6 ± 4.9	3.074 ± 0.8
CC	12 ± 1.9	9.4 ± 0.3	78.3 ± 2.2	47.6 ± 2.5	9.207 ± 0.2
CSC	25 ± 1.4	17.3 ± 1.4	69.2 ± 5	134.1 ± 15	14.094 ± 0.9
C	5 ± 0.1	3.4 ± 1	68 ± 2	0.1 ± 0.033	0.053 ± 0.009

^a^Total methane production yield after 7 days fermentation.

**Table 2:** Composition of the different groups.

Sample ID	Cow dung/g	Straw/g	Cabbage/g	Water/g	Total mass/g	Temperature/°C	C/N ratio	Water content	Replicates
UC	35	0	0	20	55	4 (unfermented)	8.1	0.95	3
SC	35	20	0	0	55	30	56.4	0.46	3
CC	35	0	20	0	55	30	18.9	0.88	3
CSC	35	10	10	0	55	30	30.2	0.75	3
C	35	0	0	20	55	30	8.4	0.95	3
