# Supplementary material for: Improving methane production in cow dung and corn straw co-fermentation systems via enhanced degradation of cellulose by cabbage addition
Source: Sci Rep. 2016 Sep 19;6:33628. doi: 10.1038/srep33628 (PMC5027527; doi:10.1038/srep33628)
Supplement: Supplementary Information [file srep33628-s1.pdf]

Improving methane production in cow dung and corn straw co-fermentation systems via enhanced degradation of cellulose by cabbage addition

Wenyang Wu[1§], Yong Chen[1§], Shah Faisal[1], Aman Khan[1], Zhengjun Chen[1], Zhenmin Ling[1], Pu Liu[1], Xiangkai Li[1*]

[1] MOE Key Laboratory of Cell Activities and Stress Adaptations, School of Life Sciences, Lanzhou University, Lanzhou, Gansu, P.R. China

[*]Corresponding Author

E-mail: xkli@lzu.edu.cn

Tel: 86-931-8912561

Fax: 86-931-8912560

[§]These authors contributed equally to this work.

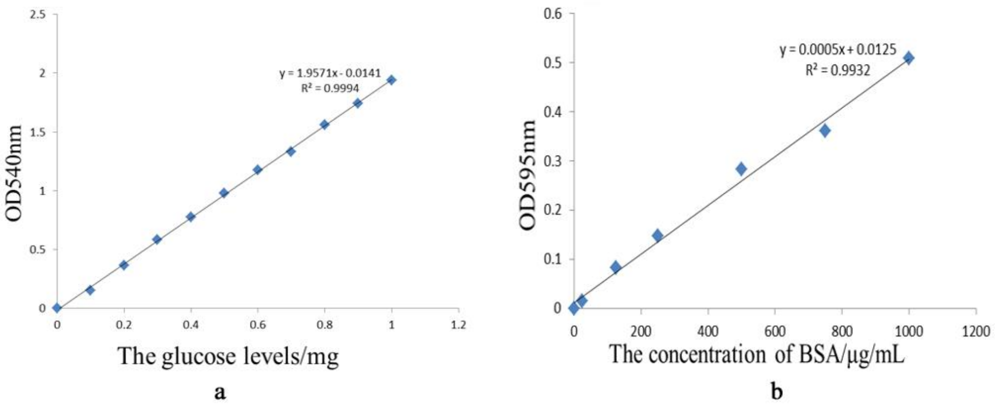

Figure S1. Standard curves of (a) glucose levels and (b) BSA concentration.

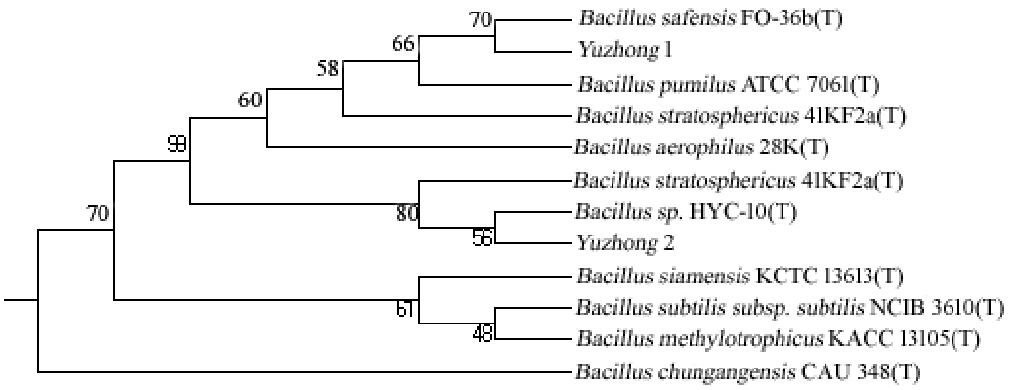

Figure S2. Phylogenetic tree of type species based on 16S ribosomal RNA sequences. The sequences were aligned using MEGA6.0. The two isolations was named Yuzhong 1 and Yuzhong 2, respectively.

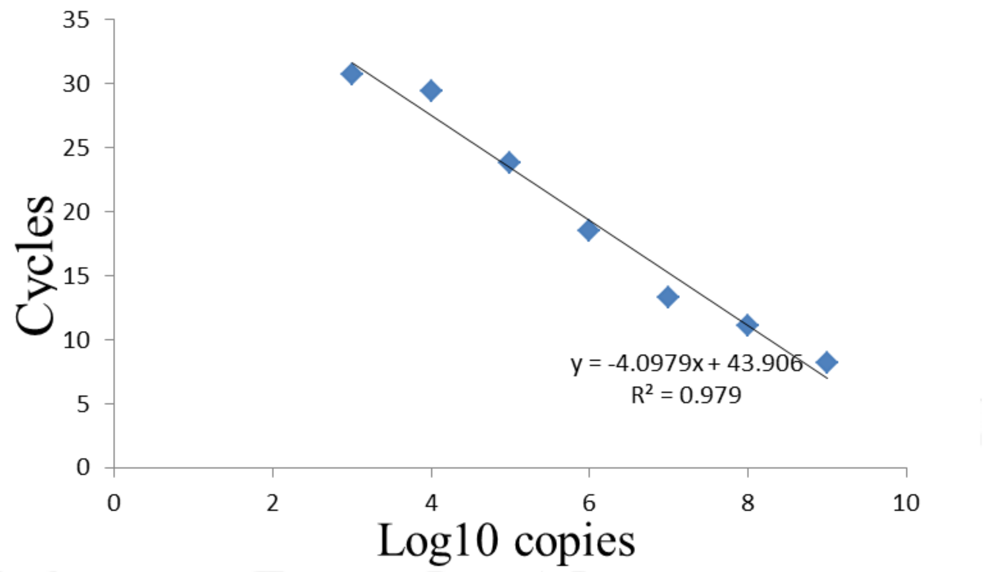

Figure S3. Standard curves of real-time PCR assay.

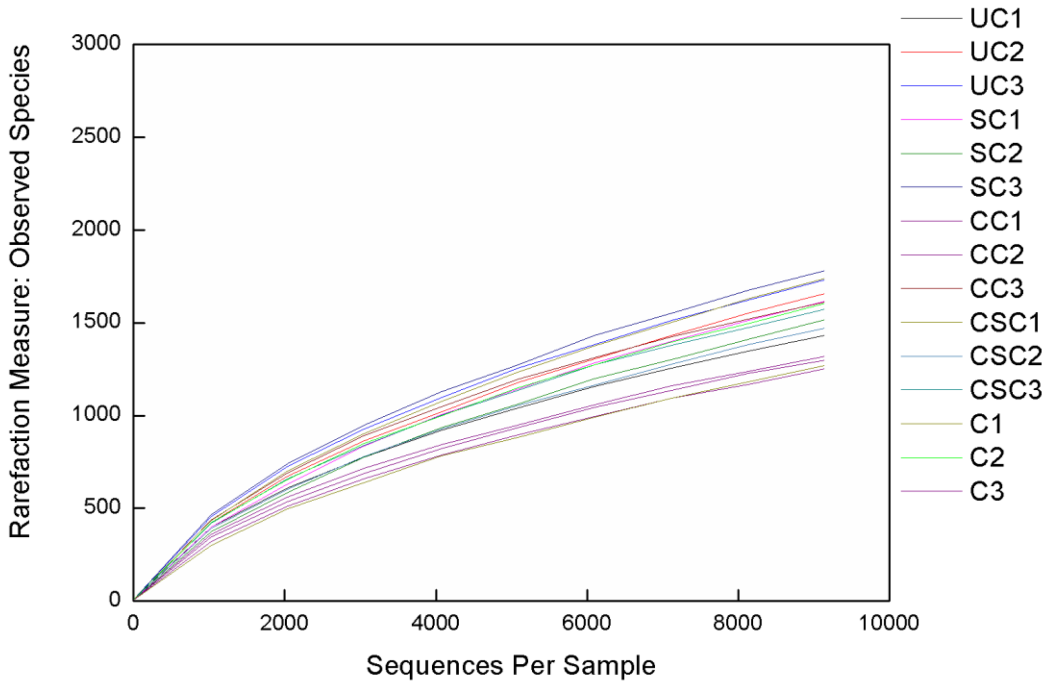

Figure S4 Rarefaction curve of different groups (97% similarity).

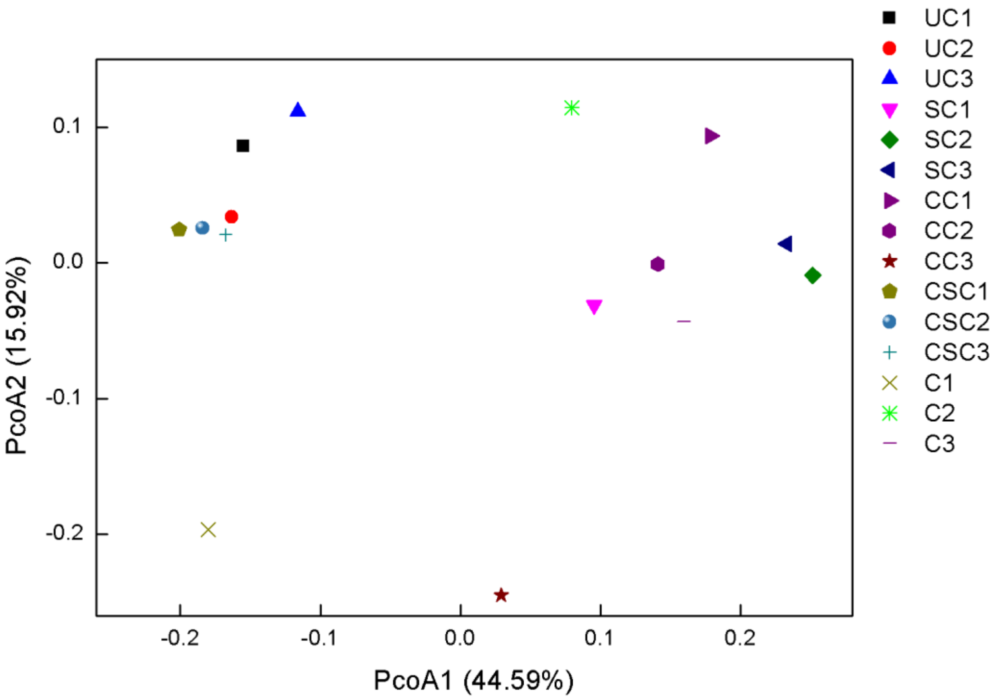

Figure S5 Principal coordinates analysis using weighted UniFrac.

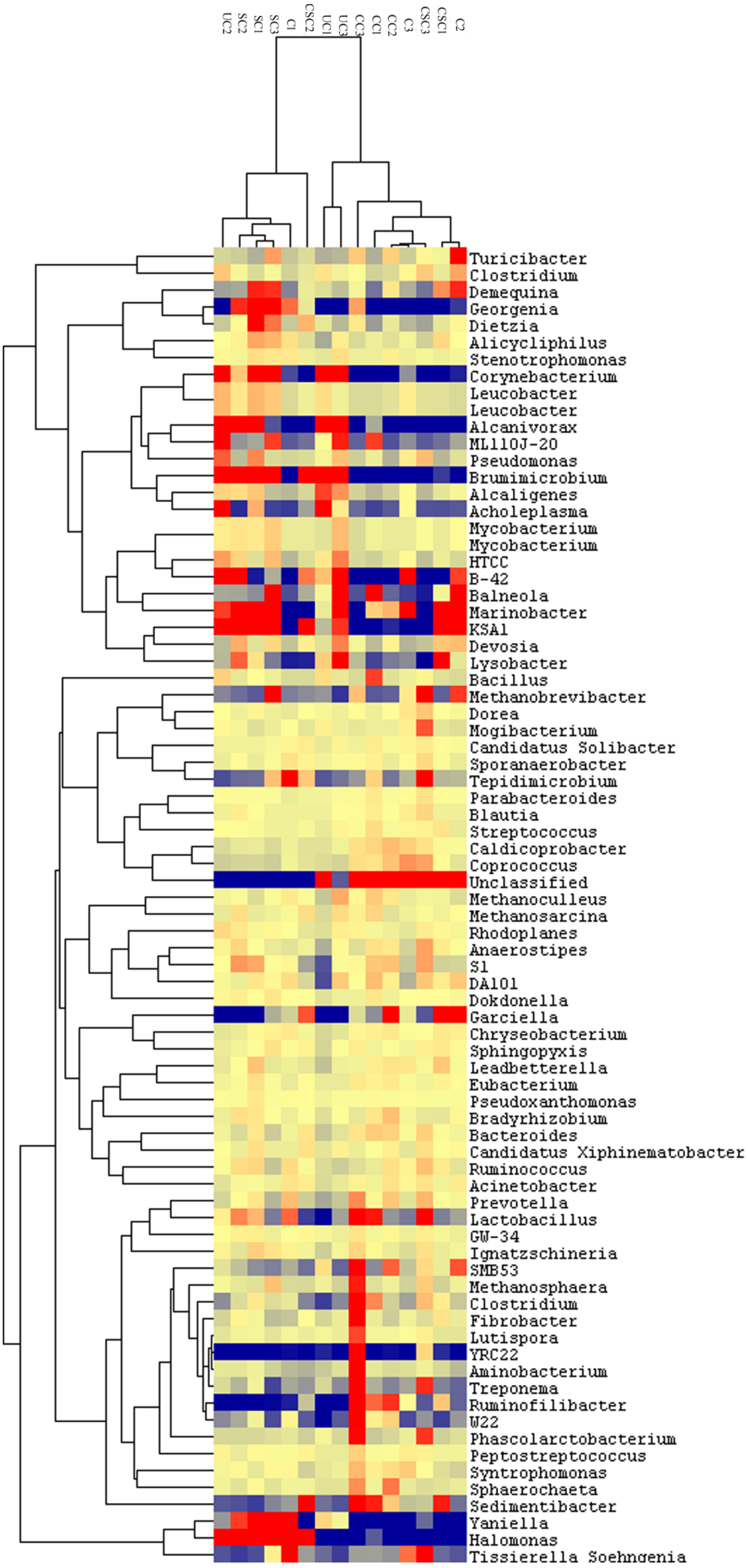

Figure S6. Heatmap.2 for microbial community clustering analysis of different treatments.

Time (d)	Biogas production (mL)				Methane content (%)			
	C	SC	CC	CSC	C	SC	CC	CSC
1	-	3±0.8	5.67±3.9	11.2±2.6	12.04±2.59	22.70±0.88	20.20±2.55	21.41±3.36
2	0.1±0.001	35.7±7.6	44±6.4	55.9±1.4	16.74±2.43	27.92±1.95	32.86±2.72	35.70±2.01
3	0.2±0.01	39.7±5.6	27.3±5.4	60.3±1.2	18.53±2.57	34.08±2.68	39.71±2.93	46.57±3.37
4	0.1±0.01	20.3±2.9	17±4.5	36±1.4	18.41±2.00	36.89±2.56	44.53±3.07	52.31±2.36
5	0.1±0.002	39±14.3	16±2.9	45±7.5	21.37±1.10	42.32±3.46	51.54±0.02	65.41±2.83
6	0.1±0.01	33±7.8	3.3±1.2	35.1±13.9	24.25±2.36	56.05±2.03	52.34±5.37	66.05±3.05
7	-	26.7±5.9	0.1±0.2	25.±7	24.82±2.41	56.04±5.44	52.51±1.76	66.24±2.73
Total value	0.6±0.02	197±8.4	113±5.4	266±9.3	-	-	-	-
Mean value	-	-	-	-	17±2.9	39.4±3.6	42.1±2.1	50.4±2.2

Table S1. Summary of biogas production and methane content in the different groups during 7 days fermentation

	UC	C	SC	CC	CSC
Cellulase specific activity (U/mg)	0.154±0.004	0.166±0. 017	0. 526±0. 062	0. 454±0. 052	0. 57±0. 008
Quantity (copies/g cow dung)	10,076,030±985,756	3,767,574±157,785	3,694,356±145,684	4,754,758±267,455	6,380,984±128,567

Table S2. Summary of the cellulase specific activity and richness of cellulose-utilizing strains in the different groups.

sample ID	seqs/sample	PD whole	chao1	goods coverage	observed species	shannon	simpson
UC	9145	115.51±4.61	3116.85±108.17	0.9±0.004	1594.13±54.74	8.48±0.1	0.991±0.001
SC	9145	110.86±5.06	3129.12±159.01	0.9±0.004	1607.73±55.45	8.67±0.08	0.99±0.004
CC	9145	117.04±2.88	3124.85±55.14	0.9±0.001	1637.97±54.33	8.07±0.25	0.971±0.007
CSC	9145	105.91±4	2453.94±59.75	0.9±0.002	1394.43±78.54	7.65±0.29	0.961±0.01
C	9145	104.53±3.09	2711.33±147.11	0.92±0.004	1392.13±75.58	7.5±0.35	0.96±0.013

Table S3. Microbial community richness and α-diversity in different groups.

Taxon	UC (%)	SC (%)	CC (%)	CSC (%)	C (%)
Euryarchaeota	0.63400271	0.34711502	0.60498753	0.77844569	0.62157715
Acidobacteria	0.485502924	0.41579553	0.49312503	0.54799589	0.46635202
Actinobacteria	10.6087972	4.96750688	2.30084118	2.61507518	5.42344344
Bacteroidetes	14.5349431	19.5983614	40.8655039	37.7437395	27.1373215
Chlorobi	0.12480759	0.05883036	0.12984404	0.08847361	0.09104215
Firmicutes	11.4988921	9.81763759	20.0597737	21.5155488	18.0331841
Nitrospirae	0.01325845	0.03943521	0.01660642	0.01957969	0,02928277
Planctomycetes	0.34309447	0.72668038	0.85440723	0.19318053	0.65393402
Proteobacteria	51.1890912	51.4229208	26.013519	30.0124561	40.0113553
Spirochaetes	0.22312844	0.25183567	0.80845129	0.35351294	0.20168595
Tenericutes	0.22224897	1.10159527	0.36808772	0.61616853	0.50220236
Verrucomicrobia	0.5935137	0.70412123	0.73011	0.59575165	0.69956218
Wwe1	0.46355738	0.27137179	1.31641925	0.37329224	0.45354917
Thermi	2.28723397	2.64530801	0.39688678	0.93477132	1.32399266
Unclassified, others	6.77792781	7.63148484	5.04143693	3.61200839	4.35151519

Table S4. Bacterial community structure in the different groups via Miseq Illumina sequencing at phylum level.
